# Draft Genome Sequence of *Taylorella equigenitalis* Strain ERC_G2224 Isolated from the Semen of a Lipizzaner Stallion in South Africa

**DOI:** 10.1128/genomeA.01205-15

**Published:** 2015-10-15

**Authors:** Catherine E. May, Martin L. Schulman, Peter G. Howell, Carina W. Lourens, Johan Gouws, Christopher Joone, Mpho S. Monyai, Misha le Grange, Oliver K. I. Bezuidt, Cindy K. Harper, Alan J. Guthrie

**Affiliations:** a Section of Reproduction, Department of Production Animal Studies, Faculty of Veterinary Science, University of Pretoria, Onderstepoort, South Africa; b Equine Research Centre, Faculty of Veterinary Science, University of Pretoria, Onderstepoort, South Africa; c Department of Veterinary Tropical Diseases, Faculty of Veterinary Science, University of Pretoria, Onderstepoort, South Africa; d Centre for Microbial and Environmental Genomics, Department of Genetics, Faculty of Natural Sciences, University of Pretoria, Hatfield, South Africa; e Veterinary Genetics Laboratory, Faculty of Veterinary Science, University of Pretoria, Onderstepoort, South Africa

## Abstract

*Taylorella equigenitalis* is the causative agent of contagious equine metritis (CEM), a sexually transmitted infection of horses. We report here the genome sequence of *T. equigenitalis* strain ERC_G2224, isolated in 2015 from a semen sample collected in 1996 from a Lipizzaner stallion in South Africa.

## GENOME ANNOUNCEMENT

*Taylorella equigenitalis* is a slow-growing microaerophilic Gram-negative coccobacillus, classified in the *Burkholderiales* order and the *Alcaligenaceae* family (1). It is the etiological agent of contagious equine metritis (CEM), a highly contagious sexually transmitted infection of horses characterized in infected mares by mucopurulent vaginal discharge and various degrees of vaginitis, cervicitis, and endometritis, and it may result in temporary infertility or early embryonic death (2). In stallions, the long-term presence of *T. equigenitalis* as a colonist of the external genitalia does not cause clinical signs, and asymptomatic carrier mares have also been reported (2). CEM is a World Organisation for Animal Health (OIE)-notifiable disease and is considered part of veterinary certification for international trade purposes (2). The multilocus sequence typing (MLST) scheme for taylorellae (3) recently provided a comprehensive overview of the genetic diversity of taylorellae. To date, the genome sequences of only four *T. equigenitalis* strains have been reported (4–6), and the genome sequences of most *T. equigenitalis* sequence types (STs) remain to be characterized.

We report here the genome sequence of *T. equigenitalis* ERC_G2224, which was isolated in 2015 from a stored frozen (-80°C) semen sample collected in 1996 from an asymptomatic carrier Lipizzaner stallion from a property in Gauteng (South Africa). Sequence typing of this strain using the *Taylorella* MLST Databases (http://pubmlst.org/taylorella/) revealed its membership in the previously nonsequenced ST4, which is not linked to one of the existing clonal complexes (CC1 to CC4) (3).

The genome of *T. equigenitalis* strain ERC_G2224 was sequenced using the Ion Torrent (Life Technologies) platform. High-molecular-weight DNA was extracted, and the size, quantity, and quality were checked using previously described methods (7). The library was constructed from 1 µg of genomic DNA using the Ion Xpress Plus fragment library kit (Life Technologies). The size selection was performed on a 2% E-Gel SizeSelect gel (Invitrogen) using the 400-bp selection criterion. Fragments were not amplified during the library-building process. Template amplification was performed using the Ion OneTouch 2 system (OT2) with the Ion PGM Hi-Q OT2 kit, and the templated particles were enriched on the Ion OneTouch ES system (Life Technologies). The samples were loaded on an Ion 316 Chip version 2 and sequenced on the Ion PGM system (Life Technologies) using the Ion PGM Hi-Q sequencing kit (Life Technologies) for 400-bp chemistry. In total, 2.25 million reads (mean length, 314 bp) generated 706 Mb of data, of which 1,123,016 reads were assembled (estimated coverage, <80×) into 18 large contigs (>500 bp), giving a consensus length of 1,670,247 bp; the contigs were ordered and compared with the genome of *T. equigenitalis* strain MCE9 (accession no. CP002456) using IonGAP: integrative bacterial genome analysis for Ion Torrent sequence data, released in 2015 (http://iongap.hpc.iter.es/iongap/) (8). Annotation was added by the NCBI Prokaryotic Genome Annotation Pipeline, released in 2013 (http://www.ncbi.nlm.nih.gov/genome/annotation_prok/), yielding 1,540 genes, 38 tRNAs, and 12 rRNAs. One clustered regularly interspaced short palindromic repeat (CRISPR)/Cas loci was detected. The average G+C content of the draft genome sequences is 37.4%.

### Nucleotide sequence accession numbers.

This whole-genome shotgun project has been deposited at DDBJ/EMBL/GenBank under the accession no. LIYJ00000000. The version described in this paper is the first version LIYJ01000000.
